# Inhibitory Effects and Mechanisms of Thymol Against *Fusarium oxysporum*, a Pathogen of Stem Base Rot

**DOI:** 10.3390/metabo16090665

**Published:** 2026-09-09

**Authors:** Xiangyu Hou, Peng Huang, Jun Wang, Yangxin Chen, Benjin Li, Feng Chen

**Affiliations:** 1 Fujian Key Laboratory for Monitoring and Integrated Management of Crop Pests, Fujian Engineering Research Center for Green Pest Management, Institute of Plant Protection, Fujian Academy of Agricultural Sciences, Fuzhou 350013, China; hxy3719198@126.com (X.H.); roc_huang@yeah.net (P.H.); 13515002711@126.com (J.W.); 2 School of Computing and Information Science, Fuzhou Institute of Technology, Fuzhou 350506, China; 13685096258@163.com

**Keywords:** thymol, *Fusarium oxysporum*, stem base rot, plant-derived fungicide, mechanism

## Abstract

**Background/Objectives**: Stem base rot caused by *Fusarium oxysporum* is a devastating soil-borne disease, yet long-term chemical pesticide application has raised serious environmental and ecological concerns. Thymol, a plant-derived fungicide, demonstrates a favorable safety profile for humans and environmental systems while exhibiting broad-spectrum antifungal efficacy. This study aimed to evaluate the inhibitory effects of thymol against *F. oxysporum* and to elucidate its underlying mechanisms of action. **Methods**: The antifungal activity of thymol was assessed in vitro against *F. oxysporum* by measuring colony and mycelial growth inhibition, sporulation, and spore germination at varying concentrations. Microscopic examination was performed to observe morphological changes in hyphae. Biochemical assays were conducted to quantify soluble protein content, DNA synthesis, ergosterol levels, and fusaric acid production in treated mycelia. Dose–response data were used to calculate median effective concentrations (EC_50_) for relevant parameters. **Results**: Thymol significantly inhibited *F. oxysporum* growth, with EC_50_ values of 51.02 μg/mL for colony growth and 57.30 μg/mL for mycelial growth. Microscopy revealed abnormal, shorter, thicker hyphae with enlarged vacuoles. Sporulation and spore germination were also effectively suppressed, with EC_50_ of 39.37 μg/mL and 53.55 μg/mL, respectively. At 100 μg/mL, thymol reduced soluble protein content by 36.73%, inhibited DNA synthesis by 47.29%, decreased ergosterol content by 48.11%, and reduced fusaric acid production by 76.86%. **Conclusions**: Thymol exerts multi-targeted inhibitory effects against *F. oxysporum* through disruption of cellular structure, impairment of macromolecular synthesis, interference with ergosterol biosynthesis, and suppression of toxin production. These findings provide a strong theoretical foundation for developing thymol as a novel biopesticide for sustainable management of stem base rot.

## 1. Introduction

Stem base rot, caused by *Fusarium oxysporum*, is a soil-borne disease that is challenging to control [1,2,3]. *F. oxysporum* infects a diverse range of plants, including chrysanthemums, yams, and tomatoes, causing stem base rot. In addition, it can infect ornamental plants such as tulips, carnations, and orchids. The fungus can be seed-transmitted and produces mycotoxins, such as T-2 toxin. Thymol has been shown to inhibit its growth and is thought to reduce toxin synthesis by disrupting cell membranes and inducing oxidative stress [4,5,6]. Currently, management strategies, including soil sterilization and root irrigation, primarily rely on chemical pesticides to control diseases caused by *F. oxysporum*. These pesticides include benzimidazoles, triazoles, methoxyacrylates, and carbamates [7]. However, chemical control has not been particularly effective against *F. oxysporum*, because the pathogen can persist in soil and cause systemic infection by colonizing vascular tissues [8,9]. Most fungicides do not reach the internal vascular systems, and even systemic products fail to completely eliminate the pathogen from infected plants [9]. The pathogen can produce chlamydospores, which further exacerbate this limitation. Chlamydospores are thick-walled survival structures that can persist in soil for decades and exhibit high tolerance to widely used single-site fungicides including azoles, strobilurins, and benzimidazoles [10,11]. Moreover, resistance to benzimidazole and other fungicide classes has been documented across multiple formae speciales of *F. oxysporum*, and the long-term effectiveness of chemical approaches is steadily declining due to the emergence of resistant strains [8,9]. Moreover, the application and accumulation of these pesticides have raised serious concerns regarding soil health and environmental ecosystems. Triazoles are known to induce oxidative stress in aquatic species, persist in soil, and impair microbial activity; moreover, these chemical compounds can be absorbed by crops, enter the food chain, and eventually threaten human and organismal health [12]. Additionally, beneficial soil organisms, such as earthworms, are significantly impacted, often facing high mortality rates due to pesticide exposure [13]. The overuse of chemical pesticides also reduces the diversity and abundance of beneficial soil microorganisms, thereby leading to disruptions in soil microecological balance [14]. Strobilurin fungicides and carbamates can leach into water bodies, causing mortality among fish and aquatic plants. Consequently, chemical pesticide contamination can cause damage to entire ecosystems, underscoring the urgent need for proactive prevention and protective measures [15,16]. Given the limitations of chemical control, biological control using antagonistic microorganisms, including *Bacillus* spp., *Trichoderma* spp., and *Streptomyces* spp., has been extensively studied and proven effective against *F. oxysporum* through mechanisms such as antibiosis, nutrient competition, and induced systemic resistance [17,18,19].

Currently, several highly toxic and environmentally hazardous pesticides are being gradually phased out, while environmentally friendly biopesticides are increasingly being developed and adopted [20]. Thymol, a plant-derived fungicide and a major component of plant essential oils, is recognized for its safety to both humans and the environment. It is primarily extracted from plants in the Lamiaceae family, most notably thyme (*Thymus vulgaris*), oregano (*Origanum vulgare*), and savory (*Satureja* sp.). It is also a major constituent in the essential oils of ajwain (*Trachyspermum ammi*) seeds and Syrian oregano (*Origanum syriacum*), with some plant varieties containing up to 96% thymol in their essential oils. Recent studies have shown that thymol exhibits significant inhibitory activity against a broad spectrum of bacteria, fungi, and viruses [21,22,23]. Thymol has demonstrated excellent broad-spectrum antifungal activity against phytopathogenic fungi such as *Aspergillus flavus*, *Penicillium expansum*, *Rhizopus stolonifer*, and foodborne pathogen *Bacillus cereus* [24]. Thymol was found to suppress bacterial wilt caused by *Ralstonia solanacearum* in potatoes [25]. Moreover, according to records from the PubChem Project of the National Center for Biotechnology Information (NCBI), thymol has been widely used as an antimicrobial disinfectant in medical settings and antimicrobial additive in food products. The European Commission has further endorsed the use of thymol as a plant protection product under Regulation No 0568/2013. These findings suggest that thymol holds potential as a biocontrol agent for the management of bacterial wilt disease.

Previous studies have demonstrated that destroying cell membrane integrity and interfering with energy metabolism are its core antibacterial mechanisms [26,27,28]. Thymol, for instance, inhibits *Streptococcus iniae* by disrupting the bacterial cell wall and membrane, leading to the leakage of key intracellular substances such as nucleotides and proteins [29]. In addition, thymol can suppress bacterial proliferation by inhibiting the activity of energy metabolism and downregulating the expression of DNA replication-related genes. Similarly, against *Enterobacter sakazakii*, thymol severely disrupts the bacterial energy supply and material transport functions by inducing cell membrane hyperpolarization, intracellular pH imbalance, and a rapid depletion of ATP [30]. In comparison, thymol exhibits a specific antifungal mechanism. For instance, in *Fusarium graminearum*, thymol destroys the cell membrane integrity, leading to intracellular electrolyte leakage, and further damages mitochondrial function by collapsing membrane potential, increasing malondialdehyde (MDA) content, and inhibiting ATP synthesis. Meanwhile, transcriptomic analyses reveal that thymol specifically downregulates key genes, TRI5 and TRI6, which are essential for deoxynivalenol (DON) synthesis. As a result, thymol not only inhibits fungal growth but also reduces toxin contamination risks. These studies highlight a multifaceted antimicrobial pathway involving membrane destruction, metabolic interference, and gene regulation, emphasizing thymol’s potential as an alternative to conventional antibiotics in addressing drug-resistant bacteria and for its application in food preservation [31].

Though previous findings have demonstrated the potential of thymol as an efficient, environmentally friendly, and multifunctional biopesticide with broad prospects for use in plant protection [13,32,33], its specific antifungal mechanism against *F. oxysporum* remains poorly understood. Thus, to further elucidate the mechanism of thymol against *F. oxysporum*, this study assessed the effects of varying thymol concentrations on the physiological characteristics (e.g., sensitivity, mycelial growth, and sporulation) and biochemical metabolism (protein content, DNA content, ergosterol content, and fusaric acid production) of *F. oxysporum*. The findings provide new insights into the antifungal mechanism of thymol and establish a theoretical foundation for the development of thymol-based biopesticide formulations.

## 2. Materials and Methods

### 2.1. Test Materials

The *Fusarium oxysporum* strain FJST-3, isolated from strawberry, was provided by the Institute of Plant Protection, Fujian Academy of Agricultural Sciences. The isolate was identified as *F. oxysporum* based on internal transcribed spacer (ITS) rDNA sequence analysis. Its pathogenicity was confirmed by inoculation assays on strawberry plants, and the strain was preserved at 4 °C on PDA medium. Thymol (99.5%) was purchased from Nanjing Hengxin Chemical Co., Ltd., Nanjing, China and dissolved in anhydrous ethanol to prepare a 100 mg/mL stock solution; Potato Dextrose Agar (PDA) medium, Yeast Extract Peptone Dextrose (YEPD) liquid medium, and Richard’s liquid medium [34] were used for cultivation.

### 2.2. Effects of Thymol on Physiological Characteristics of F. oxysporum

#### 2.2.1. Effects of Thymol on Colony of *F. oxysporum*

The well-cultured *F. oxysporum* was inoculated in the center of PDA plates supplemented with thymol at concentrations of 0 (control), 25, 50, 75, and 100 μg/mL. The 0 μg/mL group received the same volume of ethanol carrier without thymol as the treatment groups, keeping the solvent concentration constant across all plates, and cultivated under constant light conditions in a biochemical incubator (Constant Temperature and Light Incubator GZX-250BSH-III; Shanghai Xinmiao Medical Equipment Manufacturing Co., Ltd., Shanghai, China) at a constant temperature of 25 °C for 7 days, with 3 replicates per treatment [23,35]. The criss-cross method was used to measure the colony diameter, and the inhibition rate (%) was calculated according to the following formula:

inhibition rate (%) = [(control colony diameter-treated colony diameter/(control colony diameter)] × 100(1)

The effective inhibitory mid-concentration EC_50_ (μg/mL) of the thymol was determined using the colony growth diameter method. The toxicity regression curve equations were calculated from the logarithm of agent concentration (*x*) versus the inhibition rate (*y*) by IBM SPSS version 27 (IBM Corp., New York, NY, USA) software.

#### 2.2.2. Effects of Thymol on Mycelial Growth and Morphology of *F. oxysporum*

Mycelial disks of *F. oxysporum* (5 mm in diameter) were taken from the edge of the cultured *F. oxysporum* colony and transferred to an Erlenmeyer flask containing 100 mL YEPD, with 5 disks in each bottle. After incubation on a shaker at 175 r/min and 25 °C for 1 day, thymol was added to achieve final concentrations of 25 μg/mL, 50 μg/mL, 75 μg/mL, and 100 μg/mL. The treatment groups and the control group (without thymol) were further incubated on the shaker for 6 days. After filtering the culture through double-layer gauze, the mycelia were collected and their morphology observed under a microscope. The mycelia were then dried in a 25 °C oven for 24 h, and weighed to determine their dry mass [36,37,38]. Each treatment was repeated 3 times, and the mycelial growth inhibition rate (%) was calculated according to the following formula:

mycelial growth inhibition rate (%) = [(control mycelial dry weight − treated mycelial dry weight)/control mycelial dry weight] × 100(2)

From the edge of the cultured *F. oxysporum* colony, mycelial disks (5 mm in diameter) were transferred into an Erlenmeyer flask containing 100 mL PDA, with 5 disks in each flask. Thymol was added to achieve a final concentration of 25 μg/mL, 50 μg/mL, 75 μg/mL and 100 μg/mL, with no drug added as a control. After incubating on a shaker at 175 r/min and 25 °C for 7 days, spores were filtered using filter paper (Whatman Grade 1: 11 µm), and spore suspensions were prepared by adjusting the volume to 100 mL with sterile water. Spore production was determined using a hemocytometer at 40× magnification under an optical microscope (Eclipse E200; Nikon Corporation Co., Ltd., Tokyo, Japan), and each experiment was repeated 3 times [36].

Using IBM SPSS version 27 software, the regression curve equation of thymol on the inhibition rate of pathogenic fungi mycelial growth was calculated based on the logarithm of drug concentration (*x*) and mycelial growth inhibition rate (*y*), and EC_50_ was determined.

#### 2.2.3. Effect of Thymol on Conidiation and Germination of *F. oxysporum*

The spore suspension of *F. oxysporum* was adjusted to 100,000 Colony Forming Units (CFU)/mL in 100 mL of sterile water, and thymol was added to achieve final concentrations of 25 μg/mL, 50 μg/mL, 75 μg/mL, and 100 μg/mL. Flasks without thymol were used as controls. The suspensions were incubated in a constant temperature incubator at 28 °C, and spore germination was counted after 14 h. The spore suspension was observed for changes at the 14-h mark. The experiment was conducted with three replicates for each treatment. The relative inhibition rates of sporulation and spore germination were calculated according to the following equations [39]:

Spore production inhibition rate (%) = [(number of spores in control- number of spores in treatment/number of spores in control)] × 100(3)

Spore germination rate (%) = [(total number of germinated spores/total number of surveyed spores)] × 100(4)

Spore germination relative inhibition rate (%) = [(control spore germination rate-treated spore germination rate/control spore germination rate)] × 100(5)

Using IBM SPSS version 27 software, the regression curve equation for the effect of thymol on the spore production and spore germination inhibition rate of pathogenic fungi was calculated based on the logarithm of drug concentration (*x*) and spore production and spore germination inhibition rate (*y*). Additionally, the effective concentration EC_50_ for inhibiting spore production and spore germination was determined.

### 2.3. Effects of Thymol on Biochemical Metabolism of F. oxysporum

#### 2.3.1. Effect of Thymol on Soluble Protein Content of *F. oxysporum*

Mycelia were treated with thymol to achieve final concentrations of 0 μg/mL, 25 μg/mL, 50 μg/mL, 75 μg/mL and 100 μg/mL, and were collected under culture conditions as per the method in Section 2.2.2. After grinding with liquid nitrogen, 0.5 g of fungal mycelium powder was weighed into a 50 mL centrifuge tube, and 10 mL of phosphoric acid buffer (0.2 mol/L, pH 7.5) was added. The tubes were centrifuged at 4 °C and 10,000× *g* for 15 min. The supernatant was collected and stored in a refrigerator at −20 °C for later use [40].

Reagent preparation: A standard protein solution (100 μg/mg bovine serum albumin) was prepared as follows. First, 25 mg of bovine serum albumin (BSA) was weighed and dissolved in distilled water to prepare 100 mL of solution. Then, 40 mL of this solution was taken and diluted with distilled water to 100 mL. For the Coomassie brilliant blue reagent, 20 mg of Coomassie brilliant blue G-250 was weighed and dissolved in 10 mL of 95% ethanol, followed by the addition of 20 mL of 85% H_3_PO_4_. The solution was diluted with distilled water to 200 mL and then filtered through filter paper.

Drawing the standard curve: Six 10 mL glass test tubes were prepared, and standard protein solution and distilled water were added according to Table 1. The solutions were mixed well, after which 5 mL of Coomassie brilliant blue G-250 was added to each tube. Each solution was thoroughly mixed and allowed to stand for 5 min. A blank control was used in test tube 0, and the absorbance was measured at a wavelength of 595 nm. A standard curve was plotted with protein concentration on the y-axis and absorbance on the x-axis, and the linear regression equation was calculated.

Determination of soluble protein: First, 1 mL of sample supernatant was taken, and 5 mL of Coomassie brilliant blue reagent was added. The solution was mixed well, allowed to stand for 5 min, and the absorbance value was measured using the standard curve method. The experiment was repeated 3 times.

#### 2.3.2. Effect of Thymol on DNA Content of *F. oxysporum* Mycelia

According to the method described in Section 2.2.2, 0.5 g of fungal mycelium powder was collected and pulverized with liquid nitrogen under culture conditions to achieve thymol final concentrations of 0 μg/mL, 25 μg/mL, 50 μg/mL, 75 μg/mL, and 100 μg/mL. A column-based fungal DNA extraction kit (Beijing Solarbio Science & Technology Co., Ltd., Beijing, China) was used to extract DNA. The purity and concentration of *F. oxysporum* DNA were measured using a UV–visible spectrophotometer (UV-1800PC, Shanghai Mapada Instrument Co., Ltd., Shanghai, China). Detection criteria: DNA should have an absorption peak at OD_260_, where an OD_260_ value of 1 is equivalent to 50 μg/mL. The OD_260_/OD_280_ ratio should be between 1.7 and 1.9, indicating that the DNA purity is acceptable.

#### 2.3.3. Effect of Thymol on Ergosterol of *F. oxysporum*

Extraction method of ergosterol: Mycelia were collected under the culture conditions of thymol final concentrations of 0 μg/mL, 25 μg/mL, 50 μg/mL, 75 μg/mL, and 100 μg/mL according to the method described in Section 2.2. The mycelia were pulverized with liquid nitrogen, and 0.5 g of the powder was placed into a 50 mL glass test tube. Ten mL of a methanol and chloroform mixture (3:1) was added to the powder and it was kept at room temperature overnight. The next day, 10 mL each of water, chloroform and 0.5 mol/L phosphoric acid buffer containing 2.0 mol/L KCl were added. After extraction, the chloroform phase was dried using a nitrogen evaporator at 60 °C. Then, 10 mL of a methanol and ethanol mixture (4:1) containing 1.4 mol/L KOH was added and the mixture was saponified at 60 °C for 1 h. Next, 10 mL of petroleum ether was added, the mixture was vortexed, and the phases were allowed to separate into layers. The petroleum ether layer was collected and dried using a nitrogen evaporator, redissolved in anhydrous ethanol, and the volume adjusted to 10 mL. The extract was passed through a 0.45 μm filter membrane. High-Performance Liquid Chromatography (HPLC) was used to detect the concentration of ergosterol using an external standard. A histogram was constructed with the final concentration of thymol used in the bioassay placed on the x-axis and the concentration of ergosterol placed on the y-axis [41].

#### 2.3.4. Effect of Thymol on Fusaric Acid Produced by *F. oxysporum*

Mycelial disks (5 mm in diameter) were taken from the edge of cultured *F. oxysporum* colonies and transferred into an Erlenmeyer flask containing 100 mL of Richard’s liquid culture medium, with 5 disks per flask. After the flasks were incubated on a shaker at 175 r/min and 25 °C for 24 h, thymol was added to achieve final concentrations of 25 μg/mL, 50 μg/mL, 75 μg/mL, and 100 μg/mL; no drug was added as a control [42]. Cultivation was continued for 15 days, and then the mycelia were filtered using double-layer gauze, and the filtrate was collected. The filtrate was extracted three times with ethyl acetate (using 35 mL of ethyl acetate for every 100 mL of culture filtrate). The extract was then concentrated to obtain a light-yellow crude extract. The concentrated extract was dissolved in 5 mL of ethyl acetate and measured at a wavelength of 268 nm using a UV–visible spectrophotometer (UV-1800PC, Shanghai Mapada Instrument Co., Ltd., Shanghai, China).

### 2.4. Statistical Methods Used in the Study

Experimental data were organized using Microsoft Excel 2023. Linear regression equations were subsequently calculated using IBM SPSS Statistics 27.0, and Duncan’s multiple range test was employed to analyze the significance of differences among datasets.

## 3. Results

### 3.1. Effects of Thymol on Physiological Characteristics of F. oxysporum

#### 3.1.1. Effects of Thymol on Colony of *F. oxysporum*

**Figure 1:**
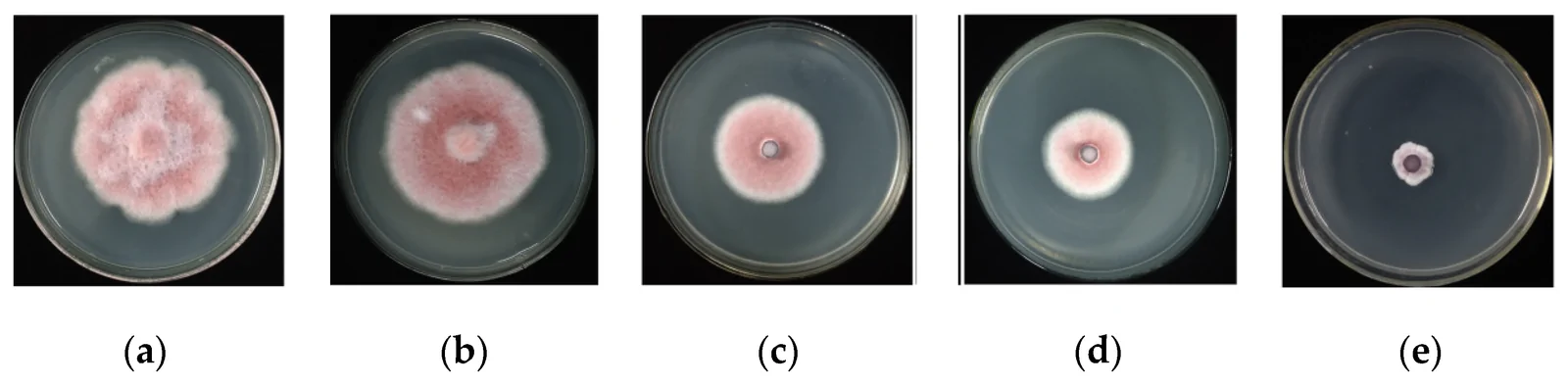
Effects of thymol on colony of *Fusarium. oxysporum*. (**a**) Control (CK) without thymol treatment; (**b**) 25 μg/mL thymol; (**c**) 50 μg/mL thymol; (**d**) 75 μg/mL thymol; (**e**) 100 μg/mL thymol.

The results indicated that thymol exerted a significant inhibitory effect on the growth of *F. oxysporum* colonies (Figure 1). The colony diameter of *F. oxysporum* decreased markedly after 7 d with the increasing concentration of thymol (Table 2). The toxicity regression curve was determined to be y = 2.21 + 4.23x, and the high correlation coefficient (r^2^ = 0.9909) confirmed the reliability of the bioassay. The half-maximal effective concentration (EC50) was calculated to be 51.02 μg/mL.

#### 3.1.2. Effects of Thymol on Mycelial Growth and Morphology of *F. oxysporum*

**Figure 2:**
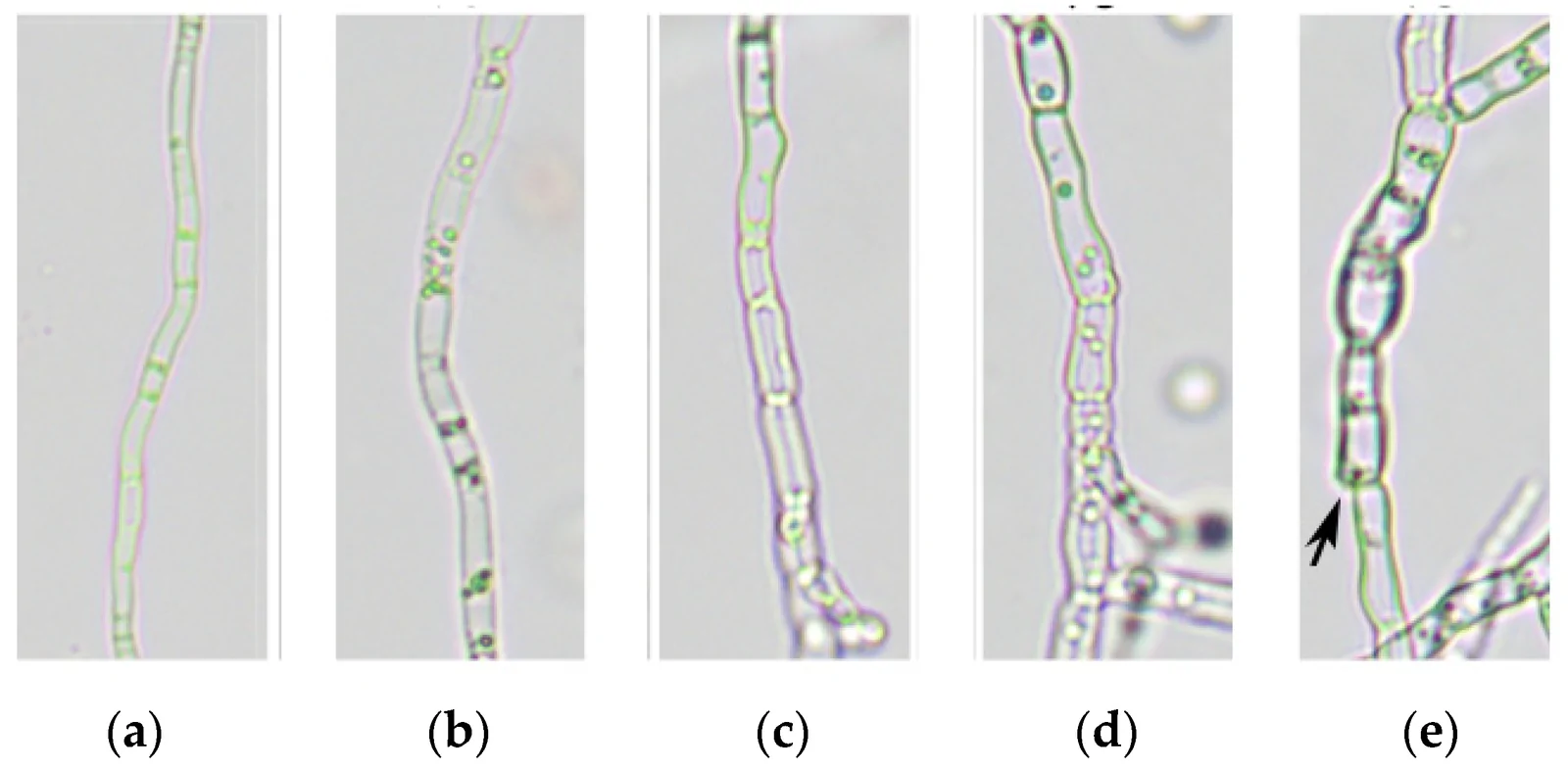
Effects of thymol on the hyphal morphology of *F. oxysporum*. Scale bar = 20 μm. (**a**) Control (CK) without thymol treatment; (**b**) 25 μg/mL thymol; (**c**) 50 μg/mL thymol; (**d**) 75 μg/mL thymol; (**e**) 100 μg/mL thymol. Arrows indicate hyphal fragmentation induced by thymol.

After liquid cultivation, the control group exhibited vigorous mycelial growth, characterized by a linear extension pattern and uniformly distributed protoplasm within the hyphal cells. In contrast, thymol treatment induced concentration-dependent morphological alterations. At a concentration of 25 μg/mL, the growth and conidial production of mycelia were inhibited, and the hyphae became noticeably shorter and thicker, with enlarged vacuoles and reduced branching as the thymol concentration increased (Table 3). At the highest concentration (100 μg/mL), mycelia growth was severely suppressed, and hyphal fragmentation was observed (Figure 2).

Based on the inhibition of *F. oxysporum* mycelial growth, a toxicity regression equation of y = 0.37 + 3.06x was constructed, with a correlation coefficient r^2^ = 0.9860, demonstrating a strong dose–response relationship. The EC_50_ value for thymol was calculated to be 57.30 μg/mL.

#### 3.1.3. Effects of Thymol on Conidiation and Germination of *F. oxysporum*

**Figure 3:**
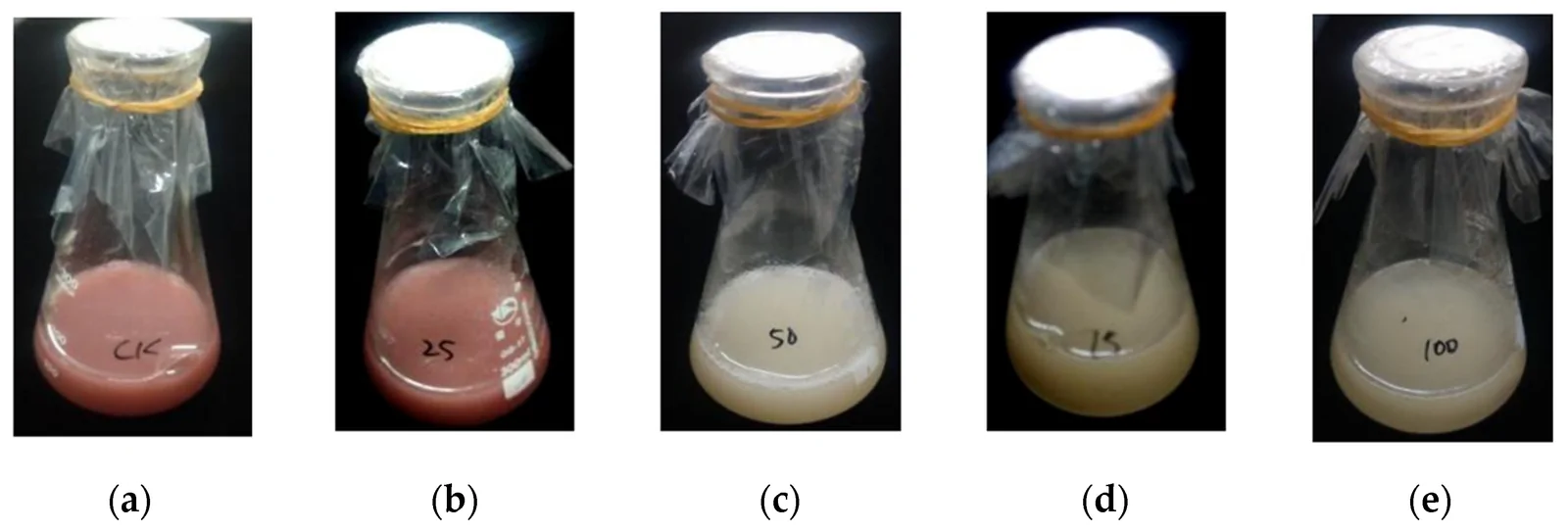
Effects of thymol on the spore germination character of *F. oxysporum*. (**a**) Control (CK) without thymol treatment; (**b**) 25 μg/mL thymol; (**c**) 50 μg/mL thymol; (**d**) 75 μg/mL thymol; (**e**) 100 μg/mL thymol.

Thymol significantly inhibited both the production and germination of *F. oxysporum* spores. The production of conidia and spore germination rate decreased markedly with increasing thymol concentration. After 14 h of incubation, the conidial production and spore germination rates in the 25~100 μg/mL thymol treatment groups were 440,000~5,552,000 CFU/mL and 13.56~89.96%, respectively. The reduction in conidial production and spore germination rates reached an inhibition rate of 94.52% and 87.63% at the highest concentration (100 μg/mL), respectively (Table 4 and Table 5). Based on the inhibition of spore production and spore germination, the EC_50_ values for thymol were calculated as 39.37 μg/mL and 53.55 μg/mL, respectively (Table 6). Additionally, after 7 d of liquid culture, a pink coloration of the medium was observed in only the control group and the 25 μg/mL thymol treatment, while no color change occurred in higher-thymol-concentration treatments (Figure 3). This result suggested that the effects of thymol on the phenotypic characteristics of *F. oxysporum* can be attributed to its inhibition of spore germination.

### 3.2. Effects of Thymol on Biochemical Metabolism of F. oxysporum

#### 3.2.1. Effect of Thymol on Soluble Proteins of *F. oxysporum*

**Figure 4:**
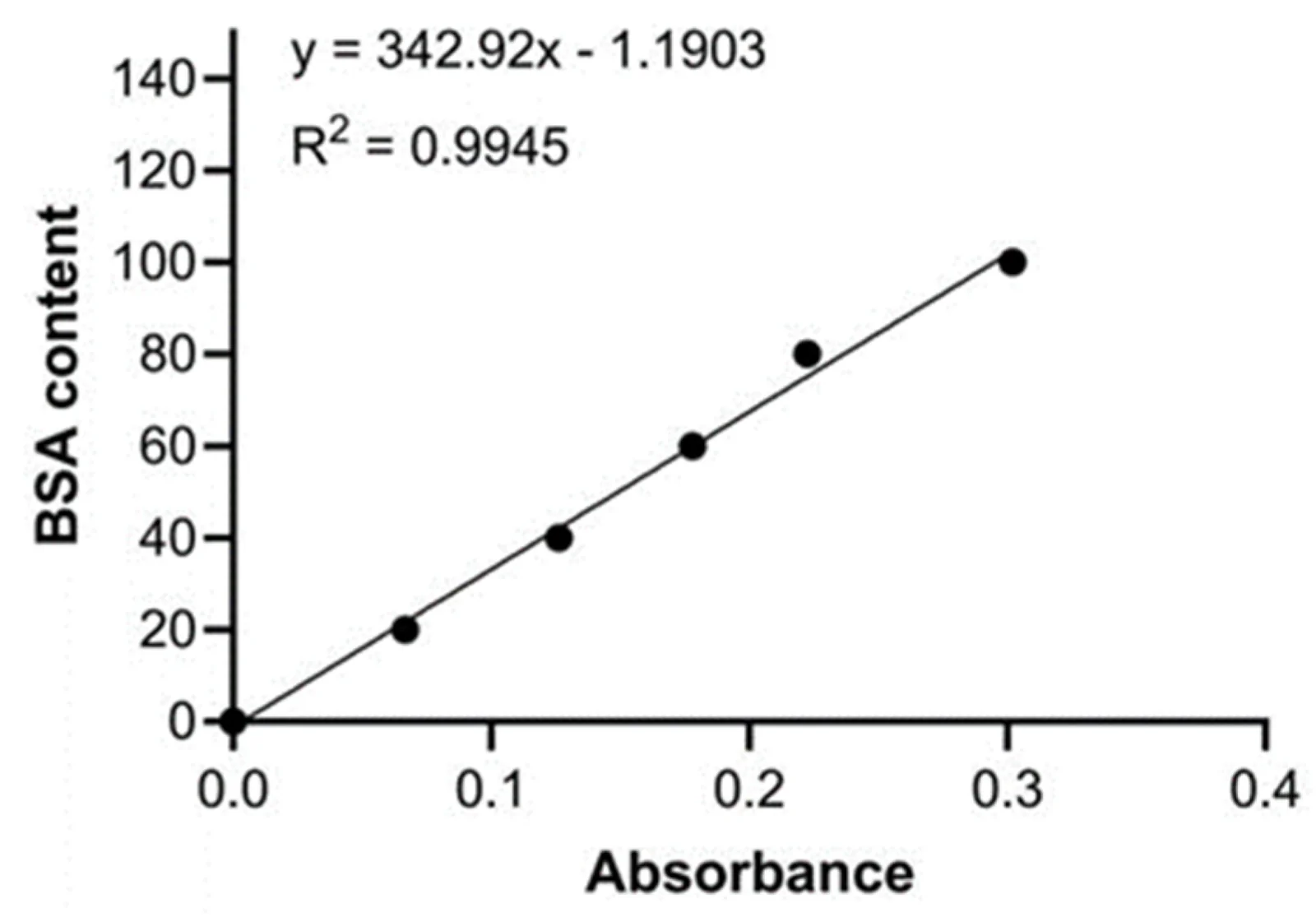
Standard curve of protein content.

The protein content of *F. oxysporum* mycelia was determined using the Bradford method, with a standard curve constructed for quantification (Figure 4). Compared to the control, the protein content of the mycelia decreased significantly with increasing thymol concentrations (Table 7). At the highest concentration (100 μg/mL), the protein content reduction corresponded to an inhibition rate of 36.73%, suggesting that thymol effectively suppresses soluble protein synthesis in the mycelia.

#### 3.2.2. Effect of Thymol on DNA Content of *F. oxysporum*

Using the ultraviolet spectrophotometer method, the DNA content of *F. oxysporum* mycelia was found to decrease significantly with increasing thymol treatment concentrations (Table 8). DNA synthesis was notably disrupted in a concentration-dependent manner, with an inhibition rate of 47.29% observed at the highest thymol concentration (100 μg/mL). These results confirm the efficacy of thymol in inhibiting DNA synthesis in the mycelia.

#### 3.2.3. Effect of Thymol on Ergosterol Content of *F. oxysporum*

**Figure 5:**
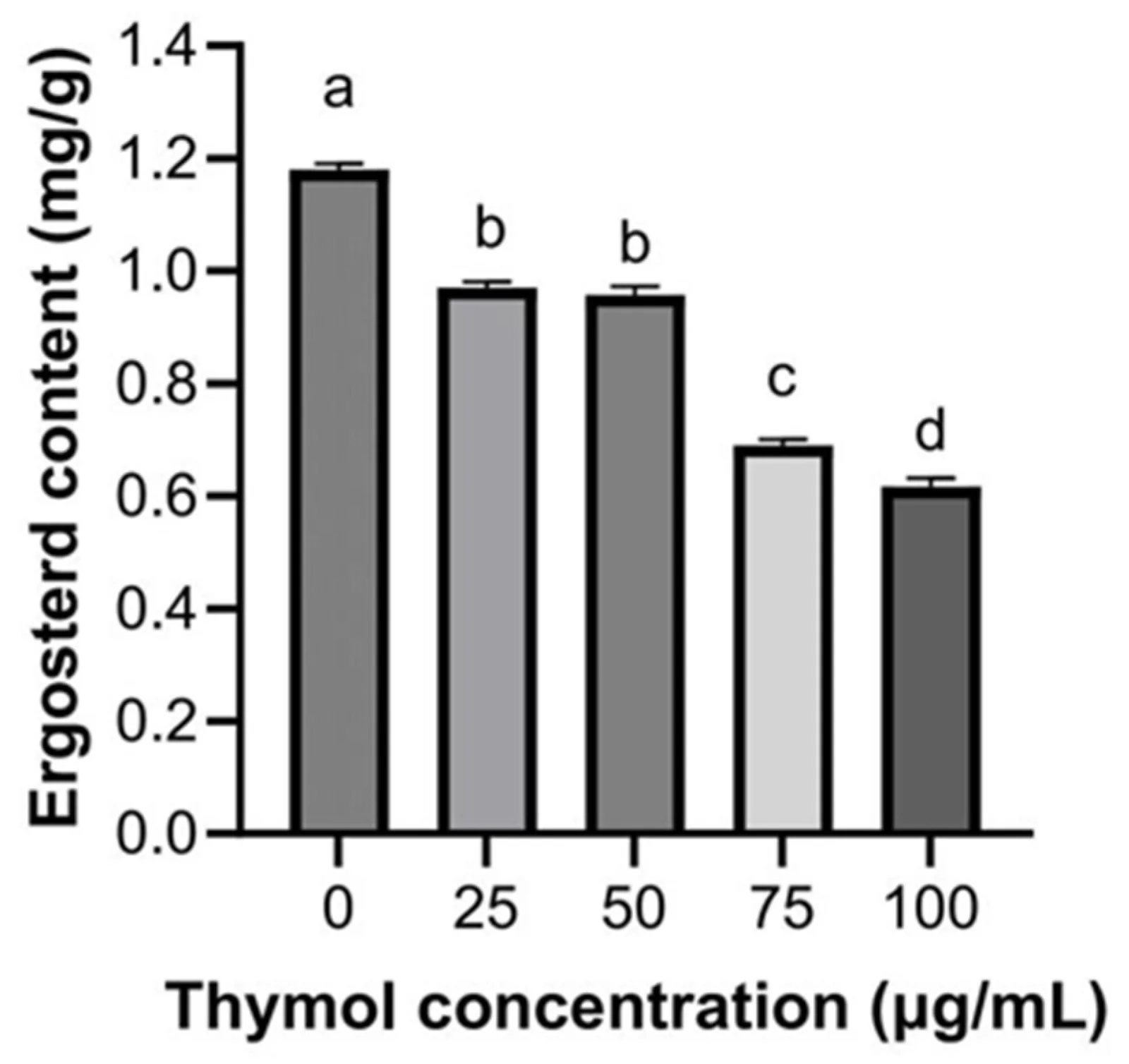
Effect of thymol on ergosterol content of *F. oxysporum*. Different letters indicate statistical significance at the level of *p* < 0.05. Data are shown as the mean ± SD. Different letters indicate a significant difference, *p* < 0.05, *n* ≥ 3.

The ergosterol content of *F. oxysporum* decreased markedly with increasing thymol concentrations (Figure 5). Compared to the control, the ergosterol content was reduced by 17.85~48.11% at thymol concentrations ranging from 25 μg/mL to 100 μg/mL, respectively. These findings indicate that thymol inhibited *F. oxysporum* by significantly reducing its ergosterol production.

#### 3.2.4. Effect of Thymol on Fusaric Acid Content in Mycelia of *F. oxysporum*

**Figure 6:**
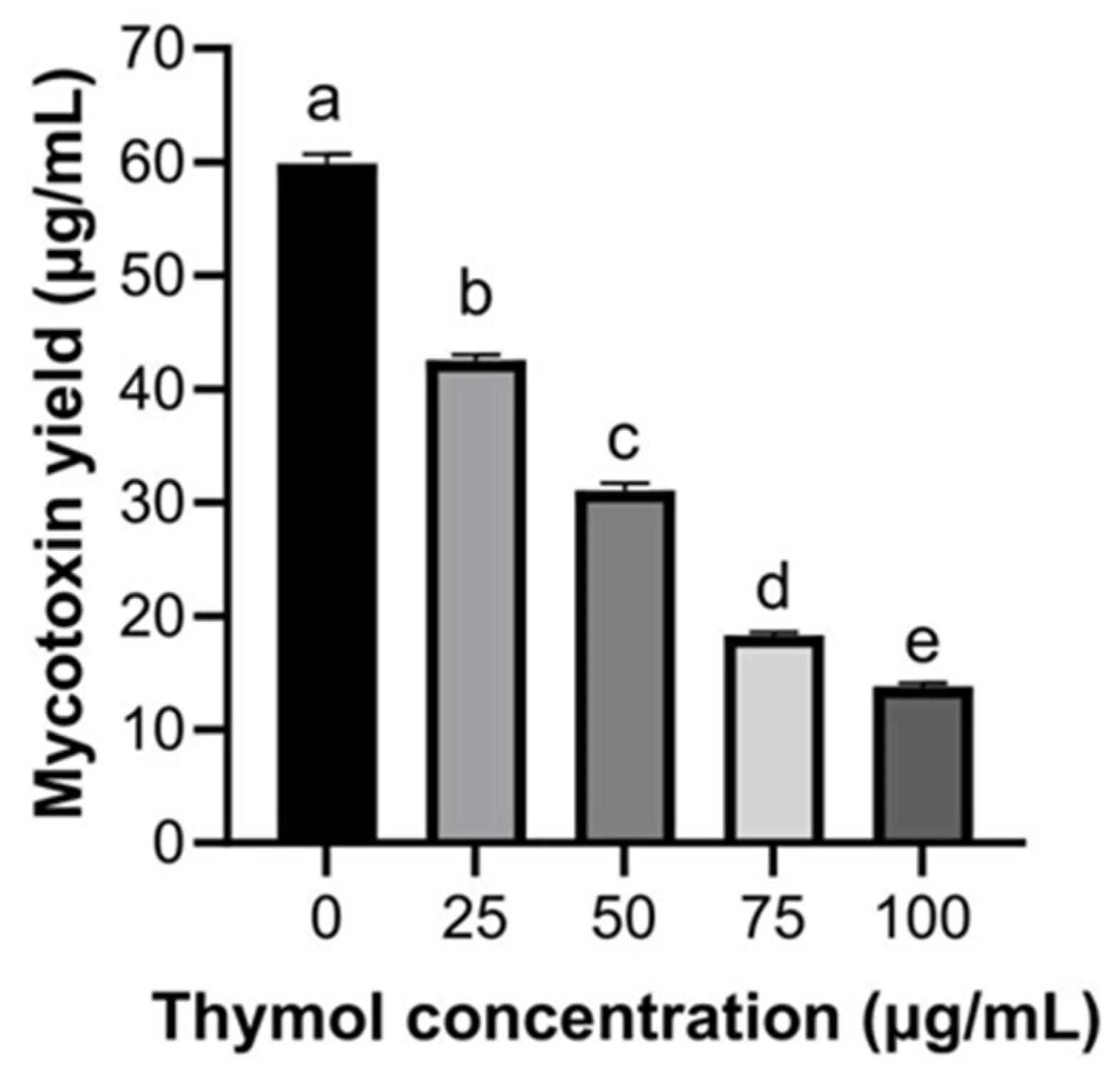
Effect of thymol on fusaric acid production of *F. oxysporum*. Data are shown as the mean ± SD. Different letters indicate a significant difference, *p* < 0.05, *n* ≥ 3.

The results indicate that the fusaric acid content in the mycelia of *F. oxysporum* decreased significantly with increasing thymol concentrations (Figure 6). Compared to the control, fusaric acid content decreased by 28.93~76.86% at thymol concentrations ranging from 25 μg/mL to 100 μg/mL, respectively. These findings suggest that thymol inhibited fusaric acid synthesis in *F. oxysporum*, thereby suppressing fungal growth.

## 4. Discussion

In this study, we evaluated the sensitivity of *F. oxysporum* to thymol and examined its effects on the fungus’s physiological and biochemical characteristics. Thymol treatment caused *F. oxysporum* hyphae to become shorter and thicker, accompanied by vacuole enlargement within the hyphae. Although the impact on germ tube morphology of conidia was not significant, thymol markedly inhibited conidial germination by inducing vacuole enlargement within conidia. These results are consistent with a previous study, which demonstrated that thymol exerts a strong inhibitory effect on both hyphal growth and spore development in *F. oxysporum* [6].

Physiological indicators, which directly reflect the growth, reproduction, and virulence of pathogens, revealed thymol’s significant inhibitory effects on the hyphal growth of *F. oxysporum.* The induced abnormal hyphal morphology was highly consistent with the previous findings in *F. oxysporum* f. sp. *niveum* [43]. Mechanistically, thymol-induced superoxide anion accumulation and oxidative damage likely explain these observed morphological abnormalities [36]. In addition to superoxide anion accumulation, thymol treatment has also been shown to trigger a cascade of oxidative stress responses, including enhanced activities of antioxidative enzymes such as superoxide dismutase (SOD) and catalase (CAT), as well as lipid peroxidation and electrolyte leakage in mycelial cells [36]. These effects collectively disrupted membrane integrity and mitochondrial function, and synergistically inhibited hyphal growth. Transcriptomic evidence further supported this by revealing downregulation of genes involved in membrane lipid synthesis and energy metabolism [6]. Moving to the biochemical level, thymol showed pronounced inhibitory effects on sporulation and conidial germination, with EC_50_ values of 39.37 μg/mL and 53.55 μg/mL, respectively. At a thymol concentration of 100 μg/mL, the conidial germination rate was reduced to just 13%, consistent with the broad-spectrum antifungal effects observed in *Fusarium graminearum* [39]. These inhibitory effects can be attributed to the ability of thymol to disrupt spore membrane integrity and to interfere with energy metabolism. In addition, the soluble protein content of *F. oxysporum* mycelia significantly decreased with increasing thymol concentration. This phenomenon can be explained by two key mechanisms: thymol downregulates the expression of ribosomal protein genes, directly inhibiting protein synthesis [39], and it induces oxidative stress that causes protein denaturation [36]. Together, these mechanisms constitute the core inhibitory pathway of thymol on fungal protein functions. Regarding DNA synthesis, the inhibition rate reached 47.29% under 100 μg/mL thymol treatment. Previous studies have shown that thymol damages fungal cell membrane integrity by reducing ergosterol content [31]. Based on this, we hypothesize that altered membrane permeability could interfere with the transmembrane transport of DNA synthesis precursors, such as nucleotides. This hypothesis aligns with prior findings that plant-derived fungicides often inhibit DNA synthesis through indirect mechanisms [43].

At the molecular level, thymol-induced ergosterol reduction may be attributed to the downregulation of ERG family genes involved in the ergosterol biosynthetic pathway, as demonstrated in transcriptomic analyses of thymol-treated *Fusarium* species [6]. Furthermore, thymol has been shown to induce mitochondrial dysfunction and reduce intracellular ATP levels in fungal cells, thereby impairing the energy supply essential for maintaining membrane integrity and supporting biosynthetic processes [31,36]. Consistent with this, ergosterol, a core component of fungal cell membranes [44,45], was reduced by 48.11% under 100 μg/mL thymol treatment. This reduction may be attributed to the obstruction of ergosterol synthesis, which disrupts cell membrane structure, leading to increased membrane permeability and leakage of intracellular contents, ultimately inhibiting fungal growth. Taken together, the energy deficit caused by ATP reduction, combined with the transcriptional suppression of ERG genes, constitutes a dual-level inhibition of ergosterol production that severely compromises cytoplasmic membrane integrity and leads to cell rupture.

Moreover, fusaric acid, a key virulence toxin of *F. oxysporum* [46], was significantly suppressed by thymol. Fusaric acid is toxic to both plants and mammals, and reducing its accumulation in agricultural crops would be highly beneficial [21,42]. Therefore, our finding that thymol significantly suppressed fusaric acid production highlights the potential of thymol in mitigating the damage caused by pathogenic fungi. Additionally, research suggests that the inhibitory effect of thymol is associated with its inhibition of pyruvate dehydrogenase, which prevents the conversion of pyruvate to acetyl-CoA, thereby suppressing ergosterol biosynthesis. This disruption compromises cytoplasmic membrane integrity, leading to increased membrane permeability and subsequent cell rupture, which contributes to the bacteriostatic and bactericidal effects of thymol.

Collectively, these findings point to a multi-target inhibitory model in which thymol concurrently compromises membrane integrity, induces oxidative damage, and disrupts energy metabolism, thereby exerting synergistic antifungal effects beyond any single pathway. This multi-target characteristic is particularly advantageous, as it may reduce the likelihood of rapid resistance development compared to single-target synthetic fungicides. Despite this multi-target advantage, the risk of resistance development should not be entirely discounted. Prolonged sublethal exposure may select for adaptive responses. For instance, thymol-adapted *E. coli* developed resistance via transcriptional reprogramming, while sublethal fungicide stress induced genomic variation in *S. sclerotiorum* that may facilitate adaptive evolution [47,48]. Resistance monitoring should therefore accompany future field deployment of thymol-based products.

Although this study has elucidated the fundamental antifungal mechanism of thymol, several limitations remain. First, the efficacy and stability of thymol under field conditions have not been validated. Environmental factors (such as soil pH, temperature, and microbial interactions) are known to significantly influence the activity of plant-derived fungicides; however, these variables were not considered in the present study. Notably, thymol hydrolysis has been shown to be pH-dependent and accelerated by acidic conditions, while its soil degradation rate is negatively correlated with soil organic carbon content, with a half-life of approximately 8.4 days in tropical soils. Therefore, future studies should include pot experiments with diverse soil types and environmental regimes to establish effective application protocols. Second, the molecular mechanism was primarily inferred from physiological and biochemical evidence. A comprehensive understanding of the regulatory network requires further transcriptomics and proteomics analyses to identify key gene expression and protein–protein interactions. Third, the potential impact of thymol on both the host plant and beneficial soil microorganisms has not been evaluated, although this constitutes a crucial criterion for both agronomic applicability and environmental safety [14,49]. A recent study demonstrated that thymol exhibits low soil sorption, indicating high bioavailability, yet its inhibition of soil enzyme activities was dose-dependent and relatively moderate compared to its isomer carvacrol [50]. This concern is not merely theoretical: studies have shown that botanical pesticides, including thymol-containing formulations, can affect mycorrhizal fungi, nitrogen-fixing bacteria, and earthworms, with effects varying by application concentration and soil type. Finally, the sensitivity of different *F. oxysporum* strains to thymol was not examined; given that strain-specific resistance differentiation may affect the efficacy of thymol-based formulations, this represents an important knowledge gap. Previous studies have shown that susceptibility to thymol varies considerably across *Fusarium* species, with *F. oxysporum* and *F. avenaceum* exhibiting higher resistance to low thymol concentrations compared to *F. culmorum* and *F. graminearum*. Given this reported interspecies variability, systematic screening of multiple *F. oxysporum* strains is essential for developing reliable thymol-based formulations and predicting their field efficacy. Thus, future studies should focus on evaluating thymol’s performance and stability under diverse local environmental conditions, investigating its impact on beneficial soil microbiota, and examining the sensitivity and resistance of different *F. oxysporum* strains to thymol.

## 5. Conclusions

Our results demonstrate that thymol exerts a potent antifungal effect, significantly inhibiting fungal growth and disrupting key physiological and biochemical processes. At the physiological level, thymol significantly inhibits hyphal growth, sporulation, and conidial germination, accompanied by severe morphological abnormalities. Biochemically, its efficacy is attributable to the inhibition of protein and DNA synthesis, a significant reduction in membrane ergosterol content, and the suppression of fusaric acid, a key virulence factor; these actions disrupt fungal structural integrity, metabolic function, and pathogenicity. This study elucidates the potential mechanism of thymol against *F*. *oxysporum* under in vitro conditions, providing a theoretical basis and foundational data for the future development of thymol-based integrated management of *F. oxysporum*.

## Figures and Tables

**Table 1:** Drawing standard curve reagent volume (mL).

Reagent	0	1	2	3	4	5
100 μg/mg Bovine serum protein solution (mL)	0.0	0.2	0.4	0.6	0.8	0.0
Distilled water (mL)	1.0	0.8	0.6	0.4	0.2	0

**Table 2:** Effects of different thymol concentrations on colony diameter and inhibition rate of *Fusairum oxysporum*.

Different Concentrations of Thymol (μg/mL)	Colony Diameter (cm)	Inhibition Rate (%)
CK	6.30 ± 0.03 a	-
25	5.70 ± 0.13 b	9.58 ± 2.01 d
50	3.48 ± 0.03 c	44.76 ± 0.49 c
75	2.26 ± 0.03 d	64.13 ± 0.47 b
100	1.26 ± 0.07 e	80.00 ± 1.10 a

Different letters indicate statistical significance at the level of *p* < 0.05.

**Table 3:** Effects of thymol on the mycelial dry weight of *F. oxysporum*.

Different Concentrations of Thymol (μg/mL)	Mycelial Dry Weight (mg)	Inhibition Rate (%)
CK	3.26 + 0.07 a	-
25	2.75 + 0.11 b	15.6 ± 3.83 d
50	2.12 + 0.03 c	35.13 ± 1.14 c
75	1.12 + 0.03 d	65.58 ± 0.25 b
100	0.69 + 0.03 e	78.94 ± 1.36 a

Different letters indicate statistical significance at the level of *p* < 0.05.

**Table 4:** Effects of thymol on the conidiation production of *F. oxysporum*.

Different Concentrations of Thymol (μg/mL)	Spore Germination Number (10^5^ CFU/mL)	Inhibition Rate (%)
CK	81.11 ± 7.39 a	-
25	40.50 ± 3.70 b	30.95 ± 10.31 d
50	55.52 ± 3.70 c	49.60 ± 7.75 c
75	4.40 ± 0.40 d	80.95 ± 2.25 b
100	15.33 ± 0.61 e	94.52 ± 0.92 a

Different letters indicate statistical significance at the level of *p* < 0.05.

**Table 5:** Effects of thymol on the spore germination rate of *F. oxysporum*.

Different Concentrations of Thymol (μg/mL)	Spore Germination Rate (%)	Inhibition Rate (%)
CK	99.00 ± 0.95 a	-
25	89.96 ± 2.73 b	11.02 ± 1.31 d
50	35.84 ± 3.52 c	44.32 ± 2.34 c
75	32.46 ± 2.76 d	68.64 ± 1.64 b
100	13.56 ± 2.35 e	87.63 ± 0.92 a

Different letters indicate statistical significance at the level of *p* < 0.05.

**Table 6:** Effects of thymol on the conidiation and germination of *F. oxysporum*.

Testing Items	Toxicity Regression Equations	Correlation Coefficient r^2^	EC50 (μg/mL)
Spore production	Y = 0.42 + 3.39x	0.9567	39.37
Spore germination	Y = 1.60 + 3.82x	0.9968	53.55

**Table 7:** Effects of thymol on soluble protein content of *F. oxysporum*.

Treatment Concentration (μg/mL)	Absorbance	Protein Content (μg/mL)	Inhibition (%)
0	0.0570 ± 0.0002 a	18.26 ± 0.27 a	-
25	0.0553 ± 0.0002 b	17.50 ± 0.09 b	4.19 ± 0.34 d
50	0.0475 ± 0.0003 c	15.05 ± 0.46 c	17.58 ± 1.01 c
75	0.0434 ± 0.0004 d	13.41 ± 0.36 d	26.59 ± 0.04 b
100	0.0371 ± 0.0002 e	11.55 ± 0.35 e	36.73 ± 0.44 a

Different letters indicate statistical significance at the level of *p* < 0.05.

**Table 8:** Effects of thymol on DNA content of *F. oxysporum*.

Treatment Concentration (μg/mL)	OD260/OD280	DNA Content (μg/mL)	Inhibition (%)
0	1.81 ± 0.02 a	67.67 ± 2.56 a	-
25	1.83 ± 0.02 a	60.33 ± 1.26 b	10.83 ± 1.04 d
50	1.85 ± 0.01 a	51.67 ± 1.25 c	23.64 ± 0.14 c
75	1.82 ± 0.04 a	42.83 ± 1.52 d	36.69 ± 0.32 b
100	1.83 ± 0.01 a	35.67 ± 0.76 e	47.29 ± 1.02 a

Different letters indicate statistical significance at the level of *p* < 0.05.

## Data Availability

The data presented in this study are available on request from the corresponding authors.
